# Malaria indicator surveys demonstrate a markedly lower prevalence of malaria in large cities of sub-Saharan Africa

**DOI:** 10.1186/1475-2875-12-313

**Published:** 2013-09-10

**Authors:** Bob S Pond

**Affiliations:** 1 Independent Consultant, Portland, Oregon, USA

**Keywords:** Urban malaria, Geospatial analysis, Malaria indicator surveys, Malaria testing

## Abstract

**Background:**

One in eight sub-Saharan Africans now lives in a city with a population greater than 750,000. Decision makers require additional evidence regarding the burden of malaria in these large cities. This paper presents results from analysis of existing data from nationwide household surveys measuring malaria parasitaemia by microscopy among children six to 59 months of age in 15 countries of sub-Saharan Africa.

**Methods:**

Geo-coordinates for each survey cluster were used to determine the distance from the cluster to the centre of each of 16 large cities with populations greater than 750,000. Geo-coordinates of each site within 25 km of the centre were entered into Google Earth to obtain a satellite image of the location and determine whether it was within the boundaries of the metropolis. In the case of two countries for which survey geo-coordinates were not available, clusters located in an additional four large cities were identified based upon their designated district. Data from all sites within city boundaries were pooled together and compared to data from all rural sites within 150 km of the city centre or in the same zone of malaria endemicity.

**Results:**

Of the 20 large cities, only in Ouagadougou were more than 10% of children found to have a malaria infection. The prevalence was less than 5% for 16 of these cities. Apart from Antananarivo where both the large city and the comparison rural communities were parasite-free, the prevalence in each of the large cities was 0 to 40% of that found among children living in rural communities within 150 km of these cities or within the same zone of malaria endemicity. In 14 of the 20 large cities, all of the children living in 75% or more of the clusters were malaria parasite-free.

**Conclusions:**

Existing data from malaria indicator surveys can be used to document the substantially lower prevalence of malaria in specific large cities. These findings will help policy makers, public health programmers and clinical workers in each country to develop and promote malaria control strategies that are suited to large cities as well as to those living in smaller communities.

## Background

In 1950 there were no cities in sub-Saharan Africa with a population greater than 750,000. Today there are 51[1]. Such large cities (here defined as those with a population greater than 750,000), cover less than 0.3% of the landmass of the region [2], yet they are home to over 100 million people – roughly one in eight sub-Saharan Africans.

Several comprehensive meta-analyses have concluded that the burden of malaria is significantly lower in large cities than in rural areas of sub-Saharan Africa [2-5]. Some other publications, however, provide a contrasting impression of the problem of urban malaria, sometimes focusing on specific urban neighbourhoods such as those near to urban agriculture which have higher levels of malaria transmission [6-18]. National planning documents and proposals sometimes state simply that “malaria is endemic throughout [the country]” [19,20], or even “malaria is hyper-endemic in all parts of the country” [21] without documenting and highlighting a low risk of the disease in metropolitan areas.

Large, nationally representative household health surveys such as demographic and health surveys (DHS) [22] and multiple indicator clusters surveys (MICS) [23] are a worthwhile source of data on the health of residents of large cities. While metropolitan areas are sometimes under-sampled for these surveys, the samples nonetheless typically include a sizeable number of residents of large cities, randomly chosen with scientific probability sampling – i.e., with a known, non-zero probability of selection for each household in the country. As a consequence, survey data can be used to estimate key indicators for sub-national as well as national populations with the precision of the estimate depending upon both the sample size for any given geographic area and the effect of sample clustering [24].

Over the last five years an extraordinary investment has been made in national surveys measuring “bio-markers”. In addition to the other indicators that they measure, malaria indicator surveys (MIS) [25] collect data on malaria parasitaemia in children, typically from six to 59 months of age (hereafter referred to as “children”) and usually based upon microscopic examination of their blood smears. To specify the location of these children, most MIS also capture the latitude, longitude and altitude of each cluster of households sampled.

This article presents estimates obtained from analysis of MIS data on the prevalence of malaria parasitaemia by microscopy among children living in each of 22 large cities located in malaria-endemic countries of Africa. These statistics are compared with estimates for children living in rural communities within 150 km of each large city or within the same zone of malaria endemicity. Also presented are survey findings concerning reported malaria testing practices in these cities.

## Methods

### Data sources

Table 1 lists the datasets obtained for analysis. This includes all of the household survey datasets in the archives of Measure DHS [22], which provide data on malaria parasitaemia. Some of these surveys are formally referred to, not as MIS, but as demographic and health surveys or AIDS indicator surveys (AIS). Datasets were also obtained from national authorities in the respective countries, for the Ghana 2011 MICS, the Kenya 2010 MIS and the Zambia 2010 MIS. For countries where more than one survey had measured malaria parasitaemia, the dataset of only the most recent survey was analysed. The Cameroon MIS measured malaria parasitaemia only by rapid diagnostic test and the results are thus not comparable and were excluded from the main analysis. Otherwise, all available datasets were included in the analysis. Suitable datasets were obtained for 15 of the 50 countries of sub-Saharan Africa.

**Table 1:** Surveys measuring malaria parasitaemia with nationally representative samples in sub-Saharan Africa^i, ii^

**Survey**	**Contact for access**	**Number of children**	**Number of children examined in each large city**^**iii, iv**^
	**to the dataset**	**examined nationwide**^**iii**^	
Angola 2011 MIS	Measure DHS	3,424	Luanda (569), Huambo (35)
Burkina Faso 2010 DHS	Measure DHS	6,059	Ouagadougou (150)
Cameroon 2011 DHS	Measure DHS	5,501	Douala (234)^iii^, Yaoundé (252) ^iii^
Ghana 2011 MICS	Ghana Statistical Service	4,461	Accra (190), Kumasi (89)
Kenya 2010 MIS	Kenya National Bureau of Statistics	4,419	Nairobi (56), Mombasa (58)
Liberia 2011 MIS	Measure DHS	3,056	Monrovia (372)
Madagascar 2011 MIS	Measure DHS	6,179	Antananarivo (226)
Malawi 2012 MIS	Measure DHS	2,068	Lilongwe (158), Blantyre (161)
Mali 2010 Special DHS	Measure DHS	1,749	Bamako (146)
Mozambique 2011 DHS	Measure DHS	4,756	Maputo (336), Matola (184)
Nigeria 2010 MIS	Measure DHS	5,067	Lagos (67), Kano (51), Ibadan (31), Abuja (7), Port Harcourt (20), Kaduna (16), Benin (26), Ogbomosho (8), Onitsha (5), Aba (6), Maiduguri (5), Enugu (8), Ilorin (12), Jos (24)
Rwanda 2010 DHS	Measure DHS	4,078	Kigali (224)
Senegal 2010 DHS	Measure DHS	4,132	Dakar (209)
Tanzania 2011–12 AIS	Measure DHS	7,501	Dar es Salaam (190)
Uganda 2009 MIS	Measure DHS	4,011	Kampala (181)
Zambia 2010 MIS	Ministry of Health of Zambia	3,423	Lusaka (230)

^i^The most recent survey for each country is listed. For several countries previous surveys have also been conducted: Angola 2006–07 MIS, Kenya 2007 MIS, Mozambique 2007 MIS, Rwanda 2007–08 Interim DHS, Senegal 2006 MIS, Senegal 2008–09 MIS, Tanzania 2007–08 AIS, Zambia 2006, Zambia 2008. Malaria Indicator Surveys conducted in the Gambia in 2007/08 and 2008/09 and in Namibia in 2009 did not measure malaria parasitaemia in the largest cities of these countries.

^ii^Reports and datasets are not yet available for the following malaria indicator surveys: Benin 2011–12 DHS, Botswana 2007 MIS, Botswana 2012 MIS, Burundi 2012 MIS, Cote d’Ivoire 2012 DHS, Democratic Republic of the Congo 2013 DHS, Equatorial Guinea 2011 DHS, Eritrea 2008 MIS, Ethiopia 2007 MIS, Ethiopia 2011 MIS, Guinea 2012 DHS, Mali 2012–13 DHS, Sierra Leone 2013 MIS, Sudan 2012–13 MIS, Zimbabwe 2012 MIS.

^iii^Number of children six to 59 months of age assessed for malaria parasitaemia with microscopy. For Cameroon, only rapid diagnostic testing was performed.

^iv^Population (2010 according to UN Department of Economic and Social Affairs) greater than 750,000.

Nationwide, the number of children examined for malaria parasitaemia ranged from 1,749 in Mali to 6,179 in Madagascar. At least 50 children were examined in each of 20 of the 35 large cities located in the 15 countries for which survey data were obtained. Unfortunately, the sample for the Nigeria 2010 MIS was too small to include sufficient children living in 12 of the 14 large cities.

### Geo-spatial analysis

For the 12 datasets (13 including Cameroon) obtained from the archives of Measure DHS, as well as for the Zambia MIS dataset, the latitude, longitude and altitude were measured with Global Positioning System (GPS) devices and specified for each cluster sampled. In a document that accompanies the datasets from the archives of Measure DHS it is stated that, "In order to ensure that respondent confidentiality is maintained, we randomly displace the GPS latitude/longitude positions for all DHS, MIS, and AIS surveys. The displacement is randomly carried out so that urban clusters are displaced up to 2 km and rural clusters are displaced up to 5 km, with 1% of the rural clusters displaced up to 10 km." The displacement is restricted so that the points stay within the same district (second administrative division) as the un-displaced cluster.

Using the haversine formula [26], a spreadsheet was used to calculate the distance between each cluster and the centre of large cities in the same country. For clusters within 25 km of a large city, the geo-coordinates were entered into Google Earth to obtain a satellite image of the site. The image was first viewed at 1:25,000 or larger scale to confirm quickly whether the site was located in the “core” - within the contiguous expanse of the city and at least 1 km from the contiguous expanse of undeveloped to semi-developed land (i.e. land which is less than half covered with structures, fenced-in compounds, roads and water) outside of the city. In this way, sites were excluded which were located in narrow extensions of the large city (e.g. sites along a road leading to an adjacent town or city). If the classification of a site could not be confirmed from the large-scale view or if the site appeared to be within 1 km of an undeveloped or semi-developed area of 0.5 km or greater in diameter, then the image was viewed at 1:5,000 or smaller scale. When viewed at this scale, an urban site (as specified by the urban/rural field included with each dataset) was classified as either “core”, “peripheral” or “other urban” depending upon whether the proportion of the surface covered with structures, fenced-in compounds, roads and water (for sites on the coast) was visually assessed to be greater than half, one quarter to one half or less than one quarter, respectively. The process of classifying clusters within 25 km of each large city took from 30 to 60 minutes depending upon the number of large cities in the country and the number of clusters in or near to them. To assure that the classification of clusters was blinded, for each survey the process was completed and finalized before the geo-coordinates and the resulting location classifications were linked to the rest of the survey data.

For eleven of the sixteen large cities for which geo-coordinates were available, the point estimate of the prevalence of malaria parasitemia was higher in clusters classified as “core” than in clusters classified as “peripheral”. However, analysis showed that, without exception, the difference in the prevalence of malaria parasitaemia between core and peripheral sites of each large city was not statistically significant (the 95% confidence interval of the relative risk overlapped 1.0 except for Antananarivo, Dakar and Kigali, where the prevalence in core clusters was zero and Bamako and Maputo, where the prevalence in peripheral clusters was zero). Hence, subsequent analysis pooled together all core and peripheral sites and compared the prevalence in these sites with that found in rural sites (as specified by the urban/rural field included with each dataset) within 150 km of the city centre. Very similar results were obtained from comparisons with rural sites within 100 km and comparisons with rural sites within 75 km. However, for some surveys the rural comparison sample became too small when the radius was reduced below 150 km. For this reason, the decision was made to compare all large cities with rural sites within 150 km (with the exception of Lusaka as noted in the next paragraph).

Antananarivo, Blantyre, Kampala, Kigali, Lilongwe, Lusaka and Nairobi each lie at greater than 1,000 m altitude where cooler temperatures may inhibit malaria transmission. For each of these seven cities, a rural comparison group was selected that matched the altitude of the city. This involved restricting the comparison sites to those rural sites within 150 km of the large city and lying at elevations within the same 200-m range of altitudes as the sites within the large city. In the case of Lusaka, there was only a single rural cluster within 150 km with a matching altitude. Hence the comparison group was extended to rural clusters with a matching altitude within 300 km of Lusaka.

Geo-coordinates were not available for the clusters sampled for the Ghana 2011 MICS or the Kenya 2010 MIS. For this reason, the clusters were positioned based upon their district (second administrative level) or, in the case of Nairobi, district and altitude. In each case, the comparison group consisted of all clusters in rural communities in the same zone of malaria endemicity (coastal zone for Accra; forest zone for Kumasi; clusters in the low malaria risk area between 1,600 and 1,800 m altitude for Nairobi; clusters in the coastal area with moderate to low malaria risk for Mombasa).

### Measurement of malaria parasitaemia

All surveys, except for the Cameroon 2011 DHS, used two methods to assess malaria parasitaemia: microscopic examination of blood smears and rapid diagnostic testing (RDT). RDTs detect circulating malaria antigens, which may persist for some days after malaria parasites have been cleared from the body following successful treatment [27], hence they assess for either recent malaria infection or current malaria infection. Only results based upon microscopy were used to compare the prevalence of malaria parasitaemia. The prevalence of parasitaemia based upon RDT was used to assess the predictive power positive of a history of fever.

Microscopic examination of a well prepared thick blood smear is considered to provide the “gold standard” for assessment of malaria parasitemia. Wongsrichanalai *et al*. [28] note that “The chance of false negative results increases with decreasing parasite densities. Greater microscopist experience and increased examination time/number of microscopic fields examined reduce such an error”. The reports of each of the MIS describe the laboratory processes employed. All MIS reports note that microscopy was conducted and supervised by a well qualified national research institution. However, only 4 of the 15 MIS reports provide information to indicate that the recommended minimum of 100 microscopic fields were examined. Ten of 15 reports indicate that all slides were read by a second laboratory technician with a third reviewer settling any discrepancies.

Particularly in drier regions, the transmission of malaria varies significantly between the rainy and dry seasons. To provide for comparability between surveys, the Roll Back Malaria Monitoring and Evaluation Reference Group recommended that malaria indicator surveys be completed within 6 weeks of the end of the rainy season [29]. Data collection for most of the surveys analysed extended over parts of the dry season as well as parts of the rainy season. Seven of the surveys (Angola, Kenya, Madagascar, Malawi, Mali, Uganda and Zambia) were completed within 6 weeks of the end of the rainy season. Another two surveys (Ghana and Senegal) were completed within 2 months of the end of the rainy season. The data collection for the surveys for Burkina Faso, Liberia, Nigeria, Rwanda and Tanzania continued until 3 to 9 months after the end of the rainy season. Of those five surveys completed more than 6 weeks after the end of the rainy season, the percentage of children examined during the dry season was lower in Accra, Dakar, Dar es Salaam, Monrovia and Kumasi than in the rural comparison clusters. The percentage of children examined during the dry season was higher in Kano (100% *versus* 44%), Kigali (95% *versus* 40%), Lagos (91% *versus* 50%) and Ouagadougou (40% *versus* 18%).

### Key indicators

In cities where at least 50 children were tested for malaria parasitaemia, estimates were made of the prevalence of malaria parasitaemia among children six to 59 months of age. Data from children outside of this age range were excluded.

Given the low prevalence of malaria infection in each of these cities, analysis also focused on two other indicators that can be calculated with data reported by household informants:

• the positive predictive value of a reported history of fever in the previous 14 days for predicting current or recent malaria infection. This is a proxy indicator for assessing the reliability of presumptive diagnosis of malaria. This was estimated from MIS data by determining the percentage of children reported to have had a fever in the previous 14 days that were positive by RDT;

• the percentage of malaria cases which were laboratory confirmed. This was estimated from MIS data by determining the percentage of children with a history of fever who were reported to have had blood drawn for testing if they were treated at a health facility.

To maximize the number of observations for these analyses, the clusters from all large cities in each country were pooled together. Estimates for all urban communities (as classified by the surveying organization – a minimum population of 5,000 is typically specified) were also calculated.

### Analysis of survey data

Survey datasets were analysed using STATA software, version 8.0. Statistics were calculated using the relevant weights and taking into account the effect of cluster sampling on confidence intervals. The 95% confidence intervals of risk ratios were calculated with modified Poisson regression to adjust for cluster sampling and provide for robust estimates [30]. In each instance where the 95% confidence interval of the risk ratio did not overlap 1.0, the prevalence in the large city was judged to be significantly less than the prevalence in the comparison rural communities.

## Results

### Prevalence of malaria parasitaemia

**Figure 1:**
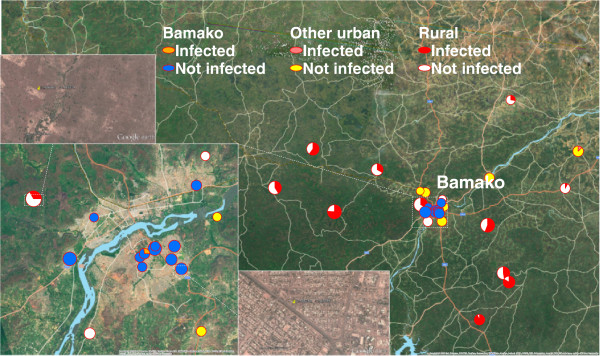
**Clusters of children six to 59 months of age who were examined by microscopy for malaria parasites.** Clusters for the Mali 2010 Special DHS within 150 km of Bamako: within Bamako *versus* other urban community *versus* rural community. For each cluster surveyed, the pie chart represents the location, the number of children examined (ten to 24) and the proportion of children infected with malaria.

Findings typical for each of the large cities are illustrated by Figure 1, which maps the results obtained for Bamako from the Mali 2010 Special DHS. Each pie chart represents a cluster of children examined. The area of each pie is proportional to the number of children examined (ranging from ten to 24) and the shaded portion of each pie indicates the proportion of children who had malaria parasitaemia. Due to the large population of Bamako (1.9 million), a large number of clusters were sampled from this metropolitan area. As shown in the inset map in the lower left of Figure 1, based upon inspection of the satellite image, 11 clusters were classified as lying in either the core or the periphery of Bamako. Ten of these clusters included zero parasitaemic children. In the remaining cluster, near to the centre of Bamako, three of 11 children were parasitaemic. Also shown in Figure 1 are 4 clusters in adjacent urban areas outside the limits of Bamako. The main map shows that there were 13 clusters in rural areas within 150 km of Bamako. The proportion of children living in these rural areas who were parasitaemic ranged from a low of one out of 12 to a high of 13 out of 14.

Table 2 summarizes findings for each of 20 large cities. Ouagadougou is the only large city having a prevalence of malaria parasitaemia greater than 10%. In fact, the prevalence was less than 5% for 16 of the 20 large cities. Apart from Antananarivo, the prevalence in the large cities was 0% (relative risk = 0.0 for Lusaka and Nairobi) to 40% (relative risk = 0.40 for Matola) of that found among children living in surrounding rural communities. The difference in the prevalence between large cities and surrounding rural communities was statistically significant for all large cities except for Antananarivo, Kigali and Mombasa -- each with a very low prevalence in the comparison rural communities. In 14 of the 20 large cities (Accra, Antananarivo, Bamako, Dakar, Dar es Salaam, Kigali, Kumasi, Lagos, Luanda, Lusaka, Maputo, Matola, Mombasa and Nairobi), all of the children living in 75% or more of the clusters were malaria parasite-free (data not shown).

**Table 2:** **The prevalence of malaria parasitaemia by microscopy among children six to 59 months living in large African cities *versus *rural comparison communities**

**Large city, country (2010 population in millions**^**i**^**)**	**Prevalence by microscopy (95% CI)**		**Relative risk = A/B (95% CI)**
	**Large city = A**	**Rural communities within 150 km**^**ii, iii **^**= B**	
Luanda, Angola (4.8)	1.4% (0.1%-2.5%)	21.4% (1.1%-41.7%)	0.07 (0.02-0.22)
Ouagadougou, Burkina Faso (1.9)	17.9% (7.5%-28.3%)	70.9% (67.8%-74.1%)	0.25 (0.14-0.45)
Accra, Ghana (2.5)	3.3% (0.5%-6.1%)	24.3% (16.2%-32.3%)^ii^	0.14 (0.06-0.34)
Kumasi, Ghana (1.9)	5.3% (1.1%-9.4)	34.7% (29.5%-40.0%)^ii^	0.15 (0.07-0.34)
Mombasa, Kenya (0.9)	0.6% (0%-2.0%)	4.7% (1.2%-8.2%)^ii^	0.12 (0.01-1.52)
Nairobi, Kenya (3.2)	0%	4.0% (0.8%-7.3%)^ii, iii^	0
Monrovia, Liberia (1.0)	7.4% (3.8%-11.0%)	31.5% (27.2%-35.8%)	0.24 (0.14-0.39)
Antananarivo, Madagascar (1.9)	0%	0%^iii^	--
Lilongwe, Malawi (0.7)	5.5% (1.8%-9.2%)	45.9% (37.1%-54.8%)^iii^	0.12 (0.06-0.24)
Blantyre, Malawi (0.7)	4.6% (1.5%-7.8%)	39.7% (20.2%-59.1%)^iii^	0.12 (0.05-0.27)
Bamako, Mali (1.9)	2.4% (0%-7.3%)	53.3% (39.6%-67.1%)	0.05 (0.01-0.34)
Maputo, Mozambique (1.1)	2.2% (0.1%-4.1%)	12.3% (5.7%-18.9%)	0.18 (0.06-0.50)
Matola, Mozambique (0.8)	4.9% (1.4%-8.4%)	12.3% (5.6%-19.0%)	0.40 (0.16-0.97)
Kano, Nigeria (3.3)	5.9% (0%-13.7%)	48.8% (35.7%-62.0%)	0.12 (0.03-0.47)
Lagos, Nigeria (10.8)	1.5% (0%-4.2%)	50.0% (25.6%-74.4%)	0.03 (0.01-0.20)
Kigali, Rwanda (1.0)	0.5% (0%-1.4%)	2.8% (1.6%-4.0%)^iii^	0.18 (0.03-1.20)
Dakar, Senegal (2.9)	0.4% (0%-1.3%)	4.5% (0.8%-8.3%)	0.10 (0.01-0.80)
Dar es Salaam, Tanzania (3.4)	0.3% (0%-1.0%)	3.9% (0.7%-7.1%)	0.09 (0.01-0.77)
Kampala, Uganda (1.6)	4.9% (1.0%-8.8%)	50.0% (40.9%-59.1%)^iii^	0.10 (0.04-0.22)
Lusaka, Zambia (1.7)	0%	10.8% (5.7%-16.0%)^iii^	--

^i^United Nations, Department of Economic and Social Affairs, Population Division (2012).

^ii^For Accra, Kumasi, Nairobi and Mombasa, the comparison group consists of clusters in rural communities in the same zone of malaria endemicity regardless of distance from the city.

^iii^For Antananarivo, Blantyre, Kampala, Kigali, Lilongwe and Nairobi, the comparison group was restricted to rural communities at an altitude matching that of the large city and within 150 km or, for Antananarivo and Nairobi, in the same zone of malaria endemicity. For Lusaka, the comparison group included all rural communities within 300 km with a matching altitude.

The prevalence of malaria parasitaemia among children in Yaoundé and Douala, where parasitaemia was measured only by RDT, was 11.8 and 11.5%, respectively. The relative risk of malaria parasitaemia among children living in these two cities compared to children living in rural communities within 150 km 0.22 (95% CI: 0.14–0.34) and 0.27 (95% CI: 0.17–0.41), respectively.

### Positive predictive value of a history of fever for diagnosing malaria

While a history of fever may often be strongly associated with malaria parasitaemia in rural areas of malaria-endemic countries, this is not the case in most urban areas, as shown in Table 3. Of the ten surveys for which at least 50 relevant observations were made in large cities, only in the large cities of Nigeria (pooled together) did more than 20% of children with a history of fever in the previous two weeks have a positive RDT.

**Table 3:** Positive predictive value of a history of fever for identifying a recent malaria infection among children six to 59 months living in large cities, all urban communities and all rural communities

**Country (large cities pooled)**	**PPV = Positive predictive value of a history of fever: % of children with fever in the last 2**								
	**weeks who have malaria by RDT**								
	**Large cities**			**Urban (pop. >= 5,000)**			**Rural (pop. <5,000)**		
	**n**	**PPV**	**95% CI**	**n**	**PPV**	**95% CI**	**n**	**PPV**	**95% CI**
Angola (Luanda + Huambo)	180	1.1%	(0%-2.5%)	376	2.3%	(0.1%-4.2%)	695	24.4%	(17.6%-31.2%)
Burkina Faso (Ouagadougou)	^*^			320	51.9%	(44.2%-59.6%)	1023	83.2%	(80.3%-86.1%)
Cameroon (Douala + Yaoundé)	108	13.6%	(6.2%-21.0%)	533	31.2%	(25.1%-37.4%)	860	50.8%	(45.4%-56.2%)
Ghana (Accra + Kumasi)	^*^			277	46.1%	(36.7%-55.5%)	858	80.2%	(76.5%-83.9%)
Kenya (Nairobi + Mombasa)	^*^			79	1.2%	(0%-3.1%)	756	25.0%	(16.9%-33.1%)
Liberia (Monrovia)	110	17.2%	(5.9%-28.4%)	569	33.3%	(24.6%-42.0%)	844	56.5%	(51.6%-61.3%)
Madagascar (Antananarivo)	63	0%	--	167	2.1%	(0%-4.5%)	689	17.9%	(12.8%-23.1%)
Malawi (Lilongwe + Blantyre)	62	13.0%	(6.2%-19.9%)	139	29.1%	(14.0%-44.1%)	486	64.1%	(57.1%-71.2%)
Mali (Bamako)	50	7.0%	(0%-15.9%)	150	19.1%	(7.1%-31.1%)	548	60.1%	(52.4%-67.9%)
Mozambique (Maputo + Matola)	54	0%	--	189	21.0%	(12.4%-29.6%)	416	58.9%	(52.2%-65.6%)
Nigeria (Lagos, Kano, Ibadan, Abuja, Port Harcourt, Kaduna, Jos, Maiduguri, Benin, Enugu, Onitsha, Ilorin + Ogbomosho)	59	35.8%	(9.9%-61.8%)	408	53.4%	(42.3%-64.4%)	1207	59.9%	(54.2%-65.6%)
Rwanda (Kigali)	^*^			85	2.2%	(0%-5.1%)	535	3.8%	(1.3%-6.2%)
Senegal (Dakar)	^*^			^*^			^*^		
Tanzania (Dar es Salaam)	^*^			215	6.5%	(2.3%-10.7%)	1254	19.7%	(15.6%-23.7%)
Uganda (Kampala)	^*^			129	49.2%	(34.4%-4.0%)	1482	67.9%	(62.2%-73.5%)
Zambia (Lusaka)	^*^			224	22.9%	(11.3%-34.5%)	839	48.1%	(39.9%-56.4%)

Note: An asterisk (*) indicates that a statistic is based on fewer than 50 unweighted cases and has been suppressed.

### Reported laboratory testing for febrile illness

Even when data from large cities are pooled together, the datasets of most nationally representative household surveys include fewer than 50 observations of children living in large cities who were reported to have been treated at a health facility for febrile illness (Table 4). Fewer than 50% of such children had blood taken for testing according to urban caretakers in ten of 14 countries and rural caretakers in 14 of 15 countries.

**Table 4:** Laboratory confirmation rates for malaria among children six to 59 months living in large cities, all urban communities and all rural communities

**Country (large cities pooled)**	**Laboratory confirmation rate for malaria = % of children with fever who were tested for**								
	**malaria if treated at a health facility**								
	**Large cities**			**Urban (pop. >= 5**,**000)**			**Rural (pop. <5,000)**		
	**n**	**Rate**	**95% CI**	**n**	**Rate**	**95% CI**	**n**	**Rate**	**95%** **CI**
Angola (Luanda + Huambo)	348	65.4%	(60.4% -70.5%)	644	64.9%	(60.7%-69.1%)	842	31.3%	(25.6%-37.0%)
Burkina Faso (Ouagadougou)	^*^			419	12.6%	(8.1%-17.0%)	1167	7.8%	(5.9%-9.7%)
Cameroon (Douala + Yaoundé)	^*^			^*^			^*^		
Ghana (Accra + Kumasi)	^*^			204	29.1%	(19.7%-38.5%)	604	36.0%	(29.6%-42.5%)
Kenya (Nairobi + Mombasa)	^*^			54	43.1%	(25.1%-61.2%)	349	25.9%	(18.6%-33.1%)
Liberia (Monrovia)	73	65.0%	(51.7%-78.4%)	394	59.4%	(51.5%-67.3%)	489	52.0%	(43.0%-61.1%)
Madagascar (Antananarivo)	^*^			97	15.2%	(3.2%-27.3%)	251	15.2%	(8.1%-22.4%)
Malawi (Lilongwe + Blantyre)	^*^			91	55.2%	(45.0%-65.3%)	241	39.7%	(31.3%-48.2%)
Mali (Bamako)	^*^			68	18.7%	(10.7%-26.7%)	140	10.4%	(2.4%-18.3%)
Mozambique (Maputo + Matola)	76	50.9%	(38.4%-63.4%)	283	51.6%	(44.2%-59.0%)	446	46.1%	(40.5%-51.6%)
Nigeria (Lagos, Kano, Ibadan, Abuja, Port Harcourt, Kaduna, Jos, Maiduguri, Benin, Enugu, Onitsha, Ilorin, + Ogbomosho)	^*^			165	9.2%	(4.3%-14.1%)	415	12.5%	(7.9%-17.0%)
Rwanda (Kigali)	^*^			100	65.7%	(53.9%-77.5%)	459	40.3%	(35.4%-45.1%)
Senegal (Dakar)	^*^			^*^			73	19.2%	(7.6%-30.8%)
Tanzania (Dar es Salaam)	^*^			179	74.9%	(65.9%-83.9%)	734	30.6%	(25.8%-5.4%)
Uganda (Kampala)	^*^			99	43.2%	(35.2%-51.3%)	1058	21.2%	(17.3%-25.0%
Zambia (Lusaka)	^*^			135	27.7%	(19.5%-6.0%)	413	28.5%	(22.8%-4.3%)

Note: An asterisk (*) indicates that a figure is based on fewer than 50 unweighted cases and has been suppressed.

## Discussion

### All malaria indicator surveys show a low prevalence of malaria in large cities

Analysis of data from MIS shows that, apart from Antananarivo where both the large city and the rural comparison communities were malaria-free, the malaria prevalence in all large cities examined was substantially lower than that found in rural comparison communities. This was also true for the two large cities of Cameroon, where malaria parasitaemia was measured only with RDT.

### Limitations of this analysis

With the method used to define the boundaries of each large city, the proportion of the population living within these boundaries cannot be determined precisely. However, from the method used, the density of development (structures, fenced-in areas and roads) outside of these boundaries is roughly 75% less than that found in the core of the city. Hence, it is likely that the great majority of the estimated populations of these large cities live within these boundaries. Higher risk peri-urban neighbourhoods may well be outside of these boundaries.

As a result of the displacement of the geo-coordinates of survey clusters, some clusters have probably been misclassified (i.e., a cluster that is actually located within a large city appearing to lie outside of the developed area). Such misclassification has the effect of reducing the estimated difference in prevalence between a large city and the surrounding rural area. Hence, the estimates of relative risk are conservative.

Two factors are possibly responsible for the uniformly consistent findings:

• While Measure DHS has introduced a random error in the reported geo-coordinates, they have assured that the reported geo-coordinates stay within the same district as the actual geo-coordinates. It is also worth noting that 2 km is the *maximum* error – not the average error;

• The datasets include a field with which the surveyors have indicated whether the cluster is “urban” *versus* “rural”. This field was used to exclude from the large city pool any rural clusters that appeared to lie within the boundaries of a large city and to exclude from the rural pool any urban clusters.

While microscopy for all surveys was performed by qualified and experienced national malaria research institutions, several of the reports provided insufficient information about the methods used. In particular, only four of fifteen reports indicated that microscopists examined at least 100 fields before concluding that a slide was parasite negative. Hence, it is possible that low level parasitaemia was missed during some surveys. It may help for the Roll Back Malaria Monitoring and Evaluation Reference Group to further elaborate on the standards for documenting the processes used for microscopic examinations carried out for malaria indicator surveys.

For sixteen of the 20 large cities studied, the percentage of children examined during the dry season was lower than or equal to the percentage of children examined during the dry season in the respective rural comparison clusters. For Kano, Kigali, Lagos and Ouagadougou, a higher percentage of children were examined during the dry season than for their rural comparison clusters. Thus, the lower prevalence of malaria found in these four cities might be interpreted as due, in part, to this discrepancy in the season when children were examined. When analysis was restricted to children examined during the dry season, however, the relative risk of malaria parasitaemia (compared to children living in rural comparison clusters) was found to still be less than 1.0 for children living in Ouagadougou (0.26), Kano (0.17), Kigali (0.31) and Lagos (0.04) (data not shown). As with the relative risks calculated with the all-seasons data, the 95% confidence intervals for these estimates did not overlap 1.0 except for Kigali.

### Previous research has also shown a markedly lower burden in large cities

These results are consistent with findings from comprehensive meta-analyses of published measurements of malaria transmission [2-4], (as measured with an annual *Plasmodium falciparum* entomological inoculation rate or A*Pf*EIR) and published measurements of the prevalence of malaria parasitaemia in children [5]. These studies each found marked differences in the burden of malaria between rural and urban areas. For example, Hay *et al*. [2] estimated the A*Pf*EIR in core areas of large cities with a population greater than one million to be substantially less (19 infective bites per person per year – ib/p/a) than that found in rural areas (1,111 ib/p/a to 2,141 ib/p/a depending upon the population density of the rural area). Peri-urban areas, adjacent to large cities but having a lower population density, were found to have an intermediate level of malaria transmission (64 ib/p/a).

The dramatically reduced transmission and prevalence of malaria infection in cities has been attributed to both environmental changes acting at the neighbourhood level (i.e., loss of or pollution of breeding waters and mosquito resting habitats can reduce mosquito breeding and the lifespan of adult mosquitoes) [31] and behavioural changes at the household level (i.e., installation of screening and use of insecticides, mosquito nets and anti-malarial medications may act to control malaria) [32]. Studies by Trape [31] showed that environment changes accompanying urban development of Brazzaville led to declines in the diversity, number, survival rates and malaria infection of anopheline mosquitoes and the frequency of human biting by these vectors. Hay *et al*. [2] cited these studies in concluding that “As a general rule, cities are unhealthy for the malaria parasite.” With increased density of human populations, malaria exposure per person also decreases [33]. Thirty-five percent of households surveyed in Lusaka reported that they had benefited from indoor residual spraying (IRS) in the preceding 12 months, as compared with 15.6% of comparison rural communities. IRS is unlikely to have contributed to the observed lower risk in the other large cities studied – the percentage of households reporting IRS in the previous 12 months was less than 15%, except in Antananarivo (66.4%), Maputo (33.4%) and Matola (25.3%), and in each of these three cases the proportion of households benefiting from IRS was lower in the large city than in the comparison rural communities (data not shown).

### Yet uncertainty persists about the burden of malaria in large cities

In spite of the predominance of evidence demonstrating a markedly lower burden of malaria in large cities, review of the published literature on urban malaria suggests that significant uncertainty persists on the topic [34]. This is in large part because the estimation of the burden of malaria in large cities requires researchers to address some methodological challenges:

• the need to distinguish the epidemiology of malaria in large cities from that in peri-urban areas [6-8] or smaller cities and towns [9-11];

• the existence of some atypically higher risk neighbourhoods within large cities [12-14];

• data from presumptive diagnoses or self-diagnoses, while widely available and frequently cited [15-18], do not reliably reflect the incidence of malaria, particularly in large cities [7,35].

When research in a large city identifies few or no cases of malaria parasitaemia, publication bias may also come into play. Analysis of data from multiple MIS permits such negative findings to come to light. Other advantages of using MIS include their contemporaneous (same year and approximately the same season) measurement of malaria parasitaemia in a standard age group and their random rather than purposive selection of subjects.

Various detailed studies have documented a higher prevalence of parasitaemia or a higher transmission of malaria in specific urban micro-environments such as those near to a swamp [13], or near to sizeable areas of urban agriculture [36-38]. Such findings should not be taken as representative of the larger metropolis in which these “hot spots” are located. Studies showing an elevated malaria risk in a few specific locations do not provide compelling evidence of an emerging urban malaria crisis [39,40], but rather of the need to identify more of these exceptional, higher risk neighbourhoods.

There is good evidence demonstrating that residents of poorer households in large cities experience a somewhat higher burden of malaria [41,42], however, the influence of poverty on the incidence of malaria in large cities should not be exaggerated. One review of research findings on urban malaria suggested that “The urban poor are found to face similar health risks when compared with the general rural population” [43]. Although this statement may fairly represent the malaria risk of poor households in some peri-urban areas or poor households in some smaller cities and towns, research to date has not demonstrated the existence of sizeable subpopulations in large cities experiencing a burden of malaria comparable to that found in rural areas.

Among the 22 large cities studied for this article, Ouagadougou in Burkina Faso is a notable outlier with a malaria parasite prevalence of 18% among the 150 children in the city who were examined for the 2010 DHS. Baragatti *et al*. [44] also found an elevated prevalence of parasitaemia (22%) when they sampled 3,354 children six months to 12 years of age in eight neighbourhoods of Ouagadougou in 2004. The prevalence in each neighbourhood ranged from 9 to 32%. These percentages are higher than those found among children living in other large cities. This may be due in part to the presence within Ouagadougou of three artificial lakes [44,45]. However, even among children living in the highest risk neighbourhoods and poorest households of Ouagadougou, the prevalence of malaria parasitaemia as measured by Baragatti *et al*. was less than half of that found in rural areas of Burkina Faso during the 2010 DHS (73%). Research published to date suggests that some neighbourhoods of Bouaké [46] in Côte d’Ivoire, Cotonou [47] in Benin, Brazzaville [48] in the Congo and Kinshasa [49] in the Democratic Republic of Congo may also have higher levels of parasitaemia than typically found in large cities. It is clearly a priority to rigorously document the burden of malaria in specific cities and in specific urban neighbourhoods suspected of being at higher risk.

The Malaria Indicator Surveys conducted in Nigeria and Angola had sample sizes too small to reliably assess the prevalence of malaria in some of the large cities in these countries. Even more importantly for a country with the population of Nigeria, the sample size needs to permit reliable estimates disaggregated to at least the level of the state/province. Until now, Malaria Indicator Surveys have had smaller samples than DHS, AIS or MICS surveys (Ruilen Ren, personal communication). The 10 Malaria Indicator Surveys analysed for this study sampled an average of 205 clusters with 5,174 households. This contrasts with the 6 DHS, AIS or MICS surveys analysed which surveyed an average of 606 clusters with 13,146 households. This suggests that more funding and time may need to be allotted to Malaria Indicator Surveys so that they can generate robust estimates disaggregated to sub-national levels.

### Progress is slow with efforts to promote laboratory confirmation of malaria

The samples for nationwide household surveys include only small numbers of children living in large cities who were reported to have experienced a recent fever. This limits the usefulness of these surveys for assessing urban practices for management of febrile illness. However, the data available suggest that a history of fever has a low positive predictive value (less than 20% for nine of the ten countries for which sufficient data are available from MIS) for identifying a child with malaria infection in large cities. This is consistent with findings from other research such as the rapid urban malaria assessments [35] which showed that less than 27% of febrile illnesses seen at health facilities in four large cities (including Ouagadougou [45]) can be attributed to malaria. Given that presumptive diagnosis is so unreliable in large cities, laboratory confirmation of malaria is essential for proper diagnosis of febrile illness. The MIS data that are available suggest, however, that progress is slow in many countries with efforts to implement new case management guidelines requiring laboratory confirmation of all malaria diagnoses. If they are to be acted upon by public health authorities, such key findings warrant verification based upon review of routine health service data or special facility-based surveys [35].

### Large cities represent an important exception and deserve to be highlighted

A large and growing percentage of the populations of malaria-endemic countries live in large metropolitan areas. National planning documents and proposals should document and highlight the special epidemiology of malaria in these large cites. Review of the malaria control proposals submitted to the Global Fund to Fight AIDS, Tuberculosis and Malaria (GFATM) [50] shows that only six of the 15 countries (16 including Cameroon) featured in this analysis acknowledged in any of their proposals that malaria transmission is low in their large cities. Only three of these countries mentioned in their proposals any interventions designed for such low transmission settings: strengthened surveillance for epidemic malaria in Antananarivo, a survey to identify specific higher risk neighbourhoods in Luanda and strengthening of malaria testing practices in Dar es Salaam.

Decision makers often demand evidence from their own country. MIS help meet this need. Other evidence can come from meta-analysis of findings from published research studies (such as those available from the website of the Malaria Atlas Project [51]) and review of routine health service data.

For the Ghana Urban Malaria Study [52], stakeholders were convened to conduct a special triangulation exercise [53]. The stakeholders identified priority questions related to urban malaria. A team of analysts compiled and analysed all data available to answer these questions then met with the stakeholders to present and discuss the conclusions. The Ghana Urban Malaria Study provided decision makers with a compelling assessment of the relative burden of malaria in large cities *versus* rural areas as well as an understanding of how the burden within large cities varied with important determinants including urban agriculture and household poverty. Stakeholders then reached consensus on recommendations for improving management of febrile illness, adjusting malaria control priorities and strengthening monitoring and research.

## Conclusion

Existing data from MIS confirm findings from comprehensive meta-analyses showing a low prevalence of malaria in large cities of sub-Saharan Africa. Evidence for particular countries will help inform the development of policies, malaria control programmes and clinical practices.

## Competing interests

The author declares that he has no competing interests.
